# Continuous Endogenous Exhaled CO Monitoring by Laser Spectrometer in Human EVLP Before Lung Transplantation

**DOI:** 10.3389/ti.2022.10455

**Published:** 2022-05-31

**Authors:** Vivien Brenckmann, Raphael Briot, Irène Ventrillard, Daniele Romanini, Maud Barbado, Kevin Jaulin, Candice Trocme, Julien De Wolf, Matthieu Glorion, Édouard Sage

**Affiliations:** ^1^ Emergency Department, Grenoble-Alpes University Hospital, Grenoble, France; ^2^ Université Grenoble Alpes, CNRS, TIMC-IMAG, Grenoble, France; ^3^ Université Grenoble Alpes, CNRS, LIPhy, Grenoble, France; ^4^ Clinical Investigation Centre for Innovative Technology (CIC-IT), Grenoble-Alpes University Hospital, Grenoble, France; ^5^ AP2E Company, Aix-en-Provence, France; ^6^ Biochemistry Proteins and Enzymes Laboratory, Grenoble-Alpes University Hospital, Grenoble, France; ^7^ Department of Thoracic Surgery, Foch Hospital, Suresnes, France; ^8^ UMR 0892, Virologie et Immunologie Moléculaires, Université Versailles-Saint-Quentin-en-Yvelines, Versailles, France

**Keywords:** lung transplant, ischemia-reperfusion, *ex-vivo* lung perfusion, lung inflammation, carbon monoxide, spectroscopy, cavity enhanced laser absorption spectroscopy, gasotransmitters

## Abstract

Endogenous production of carbon monoxide (CO) is affected by inflammatory phenomena and ischemia-reperfusion injury. Precise measurement of exhaled endogenous CO (eCO) is possible thanks to a laser spectrometer (ProCeas® from AP2E company). We assessed eCO levels of human lung grafts during the normothermic *Ex-Vivo* Lung Perfusion (EVLP). ProCeas® was connected in bypass to the ventilation circuit. The surgical team took the decision to transplant the lungs without knowing eCO values. We compared eCO between accepted and rejected grafts. EVLP parameters and recipient outcomes were also compared with eCO values. Over 7 months, eCO was analyzed in 21 consecutive EVLP grafts. Two pairs of lungs were rejected by the surgical team. In these two cases, there was a tendency for higher eCO values (0.358 ± 0.52 ppm) compared to transplanted lungs (0.240 ± 0.76 ppm). During the EVLP procedure, eCO was correlated with glucose consumption and lactate production. However, there was no association of eCO neither with edema formation nor with the PO_2_/FiO_2_ ratio per EVLP. Regarding post-operative data, every patient transplanted with grafts exhaling high eCO levels (>0.235 ppm) during EVLP presented a Primary Graft Dysfunction score of 3 within the 72 h post-transplantation. There was also a tendency for a longer stay in ICU for recipients with grafts exhaling high eCO levels during EVLP. eCO can be continuously monitored during EVLP. It could serve as an additional and early marker in the evaluation of the lung grafts providing relevant information for post-operative resuscitation care.

## GRAPHICAL ABSTRACT

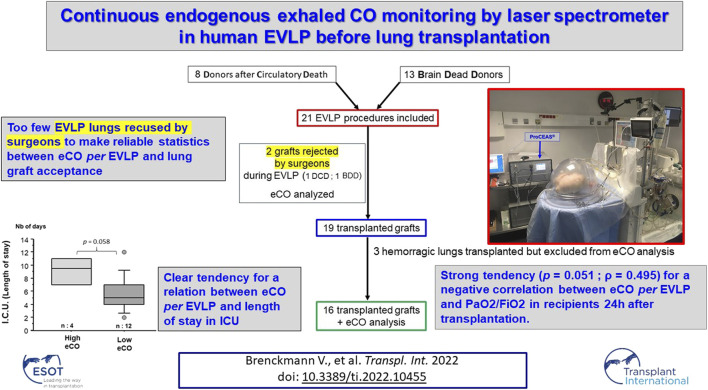

## Introduction

In an attempt to compensate for the lack of pulmonary grafts, *Ex-Vivo* Lung Perfusion techniques (EVLP) have been developed. These techniques allow a second chance to be offered in the case of certain grafts that do not initially meet all criteria for being procured for transplantation. EVLP lungs derive from either marginal donors or from donors who have undergone a controlled cardiac arrest after interruption of life-sustaining therapies (Donor after Circulatory Death (DCD) Class III of Maastricht classification). French legislation requests that lung grafts harvested from cardiac DCD donors should be evaluated by EVLP before transplantation. EVLP allows an optimization and a new evaluation of the grafts. Above all, *via* these procedures, an increase in the number of available grafts has been observed (+25% in specialized teams) with comparable long-term outcomes in recipients (1).

The lungs are subject to inflammatory phenomena, particularly to ischemia-reperfusion injury responsible for a runaway inflammatory cascade (2, 3). This will lead to lesions of the capillary-alveolar membrane up to a Primary Graft Dysfunction (PGD) associating pulmonary edema and alteration of gas exchange capacities in the recipients (4). It would be of great interest to be able to detect early severe inflammatory phenomena during an EVLP procedure, ideally by a real-time and non-invasive monitoring. Some research teams have tested the prognostic performance of dosing inflammatory proteins (cytokines) in perfusion fluids (5–7) and more specifically during the EVLP procedures (8). Overall, elevated cytokine concentrations seem to be associated with lung damage and poor recipient outcomes. Unfortunately, the real-time assay of cytokines does not seem realistic at the present time. For this reason, we aimed to monitor the production of carbon monoxide (CO) which is a biomarker of inflammation (9).

Inflammatory cytokines, *via* the MAP kinase pathway, stimulate the transcription and production of Heme-oxygenase type 1 (HO-1) (10). This enzyme will catalyze the breakdown of heme. The latter protein, present in large quantities in red blood cells, also exists in every tissue of the body, especially in the lungs (11). The breakdown of heme results in the production of biliverdin which will yield bilirubin, iron, and CO. Endogenous CO is evacuated through the respiratory tract (eCO). Many studies have already shown a correlation between pathological conditions and eCO production. For example, eCO is increased during sepsis (12), or inflammatory respiratory pathologies including allergic rhinitis, asthma, and bronchiectasis (13). More specifically, after lung transplantation, high eCO levels appear to be associated with the recruitment of alveolar neutrophils (14) and later with post-transplant bronchiolitis obliterans syndrome (13).

OF-CEAS (Optical Feedback—Cavity Enhanced Absorption Spectroscopy) is a sensitive gas concentration measurement technique based on absorption spectroscopy, on which the ProCeas^®^ instruments by the AP2E company are based. It allows very precise and real-time detection of CO with a very short response time. Its mode of operation has already been described in detail in previous publications(15, 16). Relying on healthy human volunteers, we have shown *via* the use of an OF-CEAS analyzer that breathing pure oxygen quadruples CO levels compared to ambient air conditions, whether or not the subject is a smoker (17). Additionally, our team also worked on an EVLP pig model. We proved that EVLP lungs are a source of eCO production (18), and that eCO concentrations are higher in lungs exposed to severe ischemia-reperfusion injury (19).

The main objective of this monocentric prospective study was to test, for the first time, eCO measurement by ProCeas® in an EVLP with human lungs during a real transplantation procedure in patients. We aimed to see if eCO might be an early biomarker to decide if an EVLP lung could be transplanted or not. Moreover, we compared eCO values with other evaluation parameters during EVLP procedure. We also compared EVLP eCO values with post-operative outcomes collected in transplanted recipients during the early post-operative period.

## Methods

### Design of the Study

This prospective monocentric, simple blind study of feasibility took place at Foch Hospital, Suresnes, France. Over a seven-month period (December 2018 to June 2019) twenty-one pairs of lung grafts, rejected by every French transplantation centers for a standard transplantation, were included in the protocol allowing eCO measurement during an EVLP procedure.

### Prototype “Medical ProCEAS®”

**FIGURE 1:**
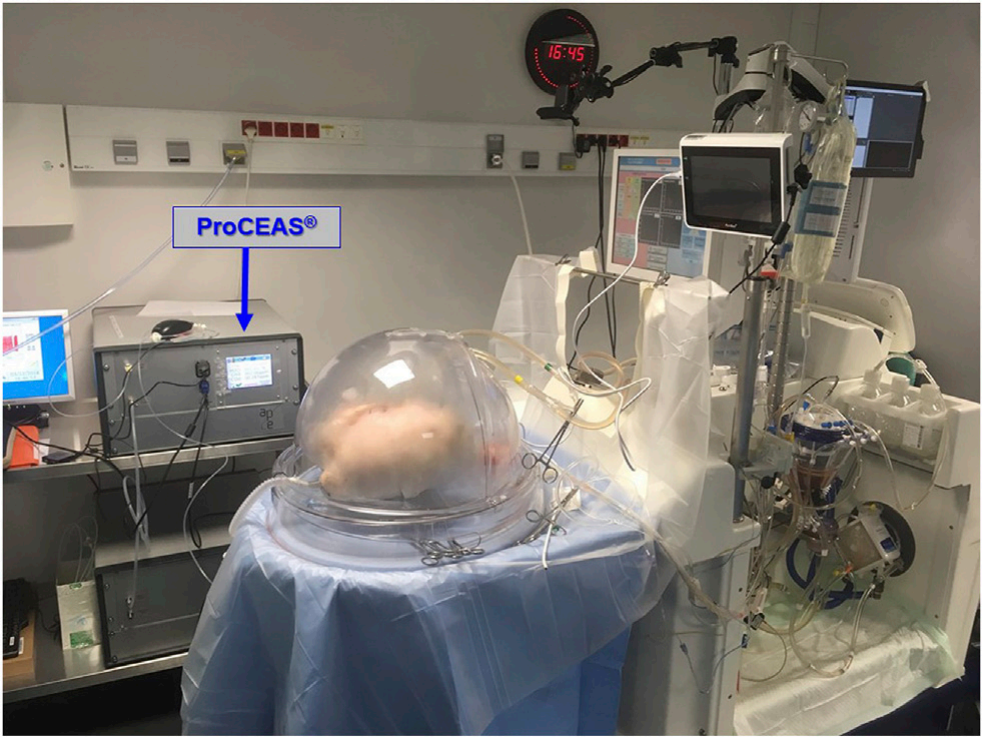
Overview of the setup of EVLP preparation with the XPS^TM^ device on the right and the ProCEAS® in the background, monitoring eCO in real-time. In the front plane is the ventilated and perfused *ex-vivo* lung.

Briefly, laser spectroscopy measurements of very low gas concentrations (less than 1 ppm) require a large light absorption path. Similar to some other spectroscopy techniques, OF-CEAS exploits a resonant optical cavity that allows an effective optical absorption path length of several kilometers while the cavity is only 1 m long (folded in two arms) and its volume about 20 cm^3^. In this way, very sensitive measurements with compact instruments and a small sampling volume can be obtained. Additionally, OF-CEAS provides absolute concentration measurements with sufficient accuracy exempting any periodic calibration with certified gas mixtures. In contrast to the somewhat complex physics underlying OF-CEAS, its optical layout consists of few basic optical elements allowing for a compact and robust device. In this study, we use an OF-CEAS analyzer prototype specially derived, for the purpose of the study, from a commercial device (ProCEAS^®^) developed by the AP2E company (Aix-en-Provence, France) one of the partners of the research consortium. This instrument, including electronics for laser control and data acquisition and a vacuum pump for sample gas circulation, measures approximately 50 × 60 × 60 cm, is transportable on a trolley and is perfectly adapted to a medical environment (Figure 1). It also connects easily with a common sterile sampling line for the ventilation on standard airway filters.

To ensure that ProCEAS® meets the constraints of safety and hygiene for its deployment in an operating room, a risk analysis has been carried out by a specialized and independent company (SurgiQual institute™; Meylan; France).

### *Ex-Vivo* Lung Perfusion Techniques

**FIGURE 2:**
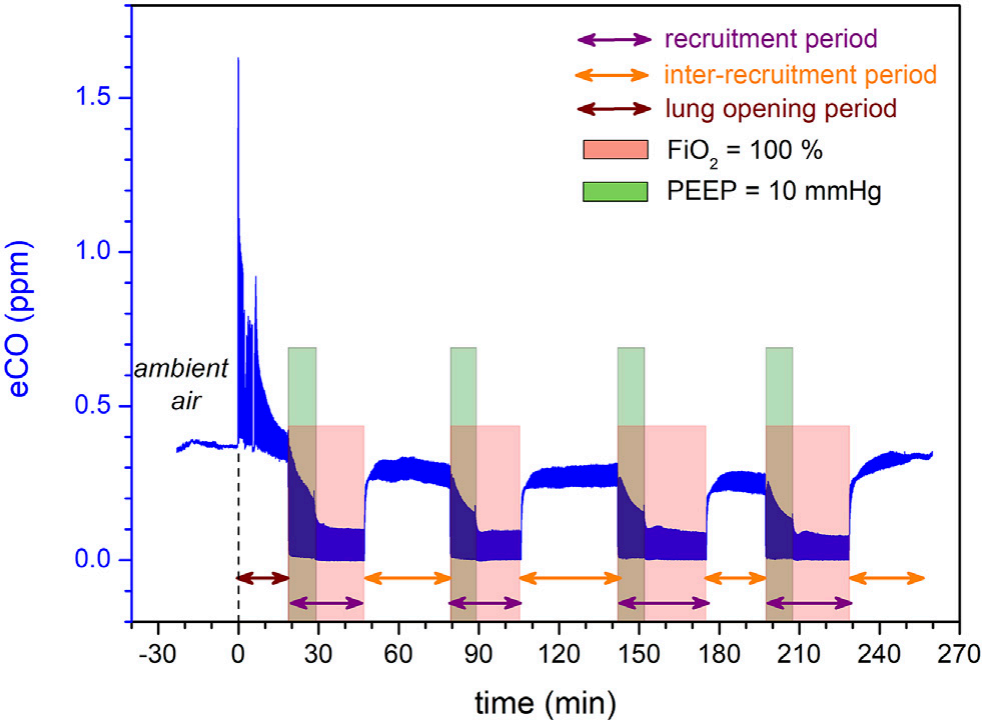
CO endogenous production monitored in real -time during *ex-vivo* lung perfusion. Time origin corresponds to the ventilation start. Before the connection to the lung, the analyzer measures CO concentration in ambient air. A strong release of eCO is observed at the beginning of the lung opening period (wine arrow). During inter-recruitment periods (orange arrows), lungs are ventilated with a FiO_2_ of 21% and a PEEP of 5 cmH2O. During each recruitment period (purple arrow), the FiO_2_ is increased to 100% (pink box), while the PEEP is increased to 10 cmH2O only during the first 10 min (green box) and then set back to 5 cmH2O.

The EVLP procedures were performed by the surgical transplant team of Foch Hospital (Suresnes; France), who perfectly masters this technique (20). Briefly, the EVLP device was an XVIVO Perfusion System (XPS shown in Figure 1, from XVIVO Perfusion AB, Göteborg, Sweden). This perfusion technique follows the “Toronto’s protocol” from the Canadian team with the greatest experience, to date, on clinical EVLP transplantation (21). The perfusion fluid was acellular (Steen® solution without red blood cells) with a target of maximum perfusate flow rate of 40% of the estimated donor cardiac output. Once the lungs reached 32°C, they started to be gently ventilated with ambient air (FiO_2_ 21%) at a frequency between 7 and 10 cycles/min, with a Positive End Expiratory Pressure (PEEP) of 5 cmH2O, and a tidal volume progressively increased up to 7 ml kg^−1^ of ideal weight. When lung perfusion and ventilation were established at their target levels (approximately 30–45 min from the beginning of the perfusion), the evaluation phase began from this steady state (Figure 2). Recruitment maneuvers were performed every hour, during which lungs were ventilated with 100% FiO_2_, first at PEEP 10 cmH2O for 10 min, and then at PEEP 5 cmH2O for others 5 min. Insufflation pressure never exceeded 25 cmH2O. Blood gases were analyzed at the end of each recruitment phase. During the inter-recruitment phases, FiO_2_ was set down to 21% and PEEP remained at 5 cmH2O. The lungs all benefited from a bronchoscopy. The decision to transplant the lungs or not was taken by the surgeon without knowing the eCO values measured by ProCEAS®.

### Data Collection

ProCEAS® was connected to the airways from the beginning of the procedure, and data were recorded continuously to the end of EVLP (Figure 1). ProCEAS® sampled small quantities of gas (200 ml min^−1^) extracted from the ventilation circuit. The eCO values were given by the difference between the maximum values (end of expiratory phase) and the minimum values (end of inspiratory phase). This made it possible to measure the production of eCO regardless of its content in the ambient air used by the ventilator when FiO_2_ was set to 21%.

ECO data were averaged over a period of 5 min following each recruitment maneuver.

Hemodynamic parameters and pulmonary mechanics (compliance, pulmonary pressure, and vascular resistance) were recorded by the XPS™ device. The quantifications of the perfusion fluids were conducted manually.

Samples of perfusate fluid were collected after each recruitment maneuver to measure blood gases, glucose, and lactate levels.

At the very end of the procedure, a sample of perfusate was taken for later cytokines measurements. The following cytokines (Interleukine (IL) 1-β, 6, 8, 10 and Tumor Necrosis Factor α (TNFα) were assayed by the MagPix device and Xponent software (Luminex Corp), using a porcine cytokine/chemokine magnetic bead panel (Milliplex MAP kit, Millipore Corp.), according to manufacturers’ instructions.

### Ethics

For the ProCEAS® prototype, which is not a medical device, a general risk management approach was carried out by the SurgiQual Institute^TM^ (http://www.surgiqual-institute.com/; Meylan, France), a company expert in Medical Device Development Regulation. The aim was to identify and reduce the safety and functional risks inherent to the design, as well to the use of the device in the context of the clinical investigation. This risk analysis was performed according to the standard ISO 14971 (dedicated to medical devices) and has concluded to a favorable benefit/risk ratio for the patients (and users). ProCEAS® measured eCO by sampling exhaled gas in a non-invasive manner. All eCO measurements were done on grafts initially rejected for transplantation by all French centers. For this study, EVLP procedures on these rejected grafts and eCO measurements were performed exclusively at Foch hospital (Suresnes, France). Surgeons could not access the measured eCO values during the EVLP, not to be influenced in their decision to requalify or not the EVLP lungs for a possible transplantation in a patient. The retrospective analysis of data from patients transplanted with EVLP lungs at Foch hospital (Suresnes; France) has been approved by the ethical committee of the French Society of Thoracic and Cardio-Vascular Surgery (approval reference: CERC-SFCTCV-2020-05-06-05-SAED).

### Statistics

The experimentation constituted a feasibility study without prior hypothesis of the eCO threshold used to get a power of the study and calculate a number of patients to include.

Our goal was to include about twenty consecutive procedures for this first study, whether the lungs were transplanted or not.

The comparisons between groups are presented in medians and interquartile ranges (box plots). Due to the relatively small number of included experiments, we verified that the distribution of the data did not differ significantly from a Gaussian distribution by a Kolmogorov-Smirnov test. In the same way, we checked the homogeneity of variances by Barlett’s test. The correlation between different parameters was performed by a simple linear regression and tested statistically by variance analysis (ANOVA). The comparison of the data between groups was made by a nonparametric test (Mann Withney U test). For the analysis of distribution between groups we used an exact Fischer test. We also performed a ROC curve with the hope to determine a clinically relevant eCO threshold to predict the evolution of the transplanted lungs. Differences with a *p* < 0.05 were considered as significant. These analyses were carried out using Statview software except for the ROC curve obtained with R version 3.6.1 (R Foundation for Statistical Computing).

## Results

We included 21 bi-pulmonary graft procedures from December 2018 to July 2019. The characteristics of donors and recipients are summarized in Tables 1, 2. Raw data of the study are available, as a single Table, in Supplementary Materials.

**TABLE 1:** Donor history of the 21 included EVLP lung grafts.

N°	Age	Height (cm)	Gender	Reason for EVLP	Anamnesis	Heart failure	Rescucitation duration before procurement (day)	Tracheal intubation duration before procurement (day)	X Ray	Bronchoscopy	PaO_2_/FiO_2_ (mmHg)
1	66	175	M	BDD	Post-traumatic brain hemorrhage	No	7	6	Effusion and atelectasis	Purulent secretions	310
2	57	165	F	BDD	Spontaneous hemorrhagic stroke	No	6	6	Atelectasiss and inhalation	Purulent secretions	327
3	48	171	M	DCD	Spontaneous hemorrhagic stroke	No	40	40	Atelectasis	Purulent secretions	522
4	61	164	M	DCD	STEMI and Heart failure	Yes	5	5	Effusion and atelectasis, Inhalation		346
5	47	183	M	DCD	Heart failure	Yes	5	5	Contusions and pneumothorax		360
6	66	170	M	BDD	Spontaneous hemorrhagic stroke	No	4	4	Atelectasis and condensations		318
7	59	200	M	DCD	Spontaneous hemorrhagic stroke	No	7	7	Condensations and inhalation		418
8	50	166		BDD	Heart failure	Yes	2	2	Effusion and atelectasis	Purulent secretions	322
9	52	170	M	BDD	Spontaneous hemorrhagic stroke	Yes	2	2	Contusions and inhalation	Blood	300
10	53	165	F	BDD	Post-traumatic brain hemorrhage	No	1	1	Condensations	Purulent secretions	388
11	41	180	M	BDD	Spontaneous hemorrhagic stroke	No	1	1	Inhalation	Blood	330
12	34	168	F	DCD	Spontaneous hemorrhagic stroke	No	5	5	Atelectasis		480
13	34	183	M	BDD	Spontaneous hemorrhagic stroke	No	4	4	Effusion	Purulent secretions	266
14	31	187	M	BDD	Hanging	Yes	1	1	Condensations	Blood	404
15	64	158	F	DCD	Spontaneous hemorrhagic stroke	Yes	1	1			528
16	15	185	M	BDD	Heart failure	Yes	14	14	Contusions and inhalation	Purulent secretions	353
17	21	178	M	BDD	Post-traumatic brain hemorrhage	Yes	4	4	Atelectasiss and inhalation	Purulent secretions	375
18	28	182	M	BDD	Post-traumatic brain hemorrhage	Yes	4	4	Contusions and inhalation		401
19	53	167	F	BDD	Spontaneous hemorrhagic stroke	Yes	1	1	Inhalation	Purulent secretions	277
20	66	146	F	DCD	Heart failure	Yes	10	10	Effusion and atelectasis	Purulent secretions	362
21	61	162	F	DCD	Drowing and Heart failure	Yes	7	7	Effusion and atelectasis		297

M, male; F, female; BDD, brain dead donors; DCD, donor after circulatory geath. In dark grey, the two non-accepted lungs for transplant after EVLP. The lungs 9, 11, and 14 had macroscopical blood in their respiratory tract. They were transplanted to recipients by surgeons but we excluded them from the eCO analysis.

**TABLE 2:** Medical outcomes of the 19 recipients transplanted with EVLP grafts.

N°	Indication	Age	Resuscitation duration after Tx (day)	Total Hospitalization duration (day)	PaO_2_/FiO_2_ at H0 (mmHg)	PGD at H0	PaO_2_/FiO_2_ at 24 h (mmHg)	PGD at 24 h	PaO_2_/FiO_2_ 48 h (mmHg)	PGD at 48 h	PaO_2_/FiO_2_ 72 h (mmHg)	PGD at 72 h	DPG 3 during first 72 h
1	AAT	55	4	24	206	2	440	1	413	1	380	1	No
2	CF	35	4	25	314	1	328	1	360	1	314	1	No
3	CF	20	4	17	476	1	366	1	541	1	490	1	No
4	COPD	53	12	40	114	3	166	3	204	2	171	3	Yes
5	CF	48	5	35	194	3	519	1	419	1	350	1	Yes
8	CF	36	7	24	89	3	359	1	329	1	428	1	Yes
9	COPD	57	12	33	151	3	168	3	190	3	283	2	Yes
10	CF	37	8	23	97	3	466	1	271	2	366	1	Yes
11	CF	33	4	26	312	1	400	1	271	1	324	1	No
12	CF	21	6	23	283	2	346	1	391	1	289	2	No
13	CF	39	7	22	392	1	388	1	367	1	304	1	No
14	CF	34	3	28	246	2	316	2	444	1	280	2	No
15	CF	20	2	23	437	1	437	1	357	1	440	1	No
16	CF	48	3	29	182	3	248	2	412	1	370	1	Yes
17	COPD	59	6	42	90	3	337	1	382	1	353	1	Yes
18	COPD	64	10	31	181	3	418	1	370	1	436	1	Yes
19	Fibrosis	42	9	26	ECMO	3	ECMO	3	ECMO	3	ECMO	3	Yes
20	Alveolar proteinosis	22	12	37	ECMO	3	ECMO	3	ECMO	3	ECMO	3	Yes
21	Cystic pneumonia	43	4	22	309	1	345	1	310	1	309	1	No

AAT, Alpha-1 antitrypsin deficiency; CF, cystic fibrosis; COPD, chronic obstructive pulmonary disease; ECMO, extracorporeal membrane oxygenation; PGD at HO, primary graft dysfunction score at admission intensive care unit etc.

The lungs 9, 11 and 14 (highlighted in gray), were transplanted to recipient but excluded from the eCO analysis (macroscopical blood in their respiratory tract). A subgroup analysis comparing clinical outcomes of patients transplanted with these hemorrhagic lungs versus other patients transplanted with non-hemorrhagic lungs did not show any statistical difference in the above clinical outcomes (nonparametric Mann Withney U test).

The lungs 6 and 7 were non-accepted for transplant after EVLP and are not presented in this table.

During the EVLP procedure, only 2 grafts were rejected for transplantation by the surgical team. These two rejected lungs had absorbed large amounts of perfusion fluid during the procedure; their compliance had decreased significantly and their PO_2_/FiO_2_ ratio was less than 350 mmHg.

**FIGURE 3:**
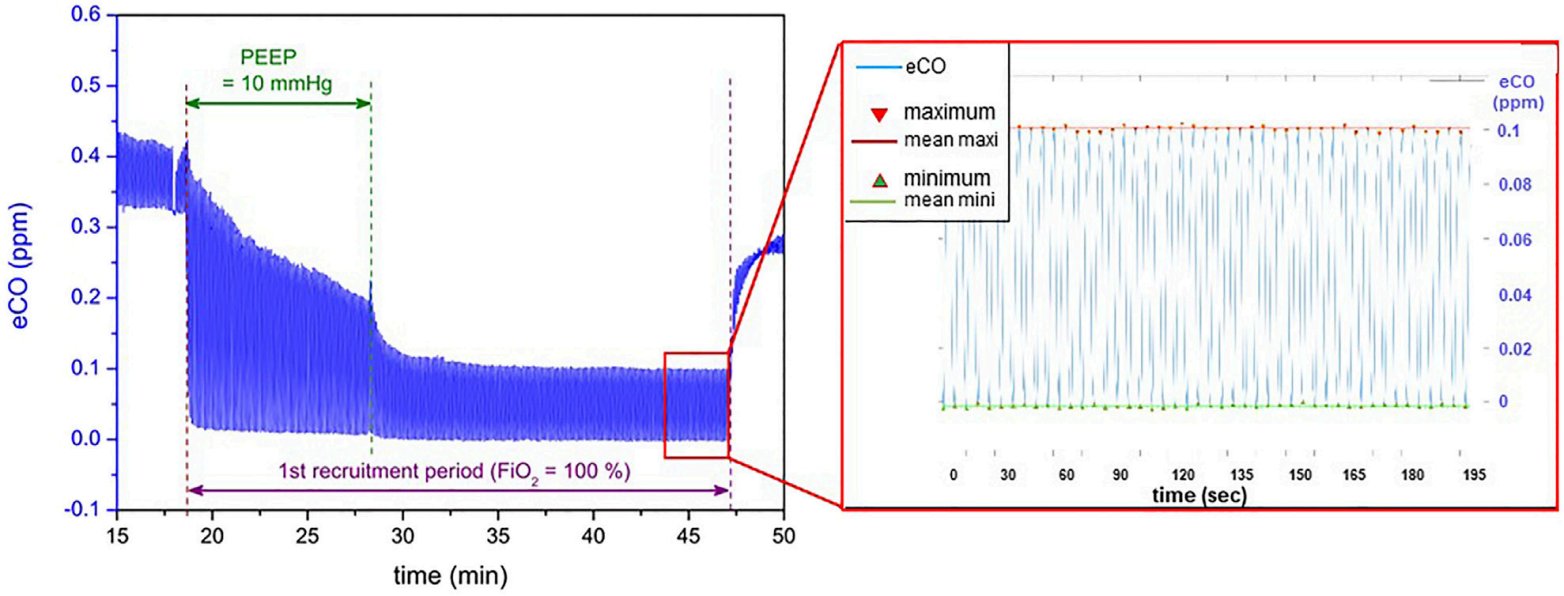
Zooms-in of eCO measurements presented in Figure 2 (Data from one EVLP procedure, displayed as example) Left: entire first recruitment period. Right: zoom on the last minutes of the recruitment phase. The respiratory cycles can be distinguished, allowing the identification of maximum (red) and minimum (green) CO values to derive the lungs’ endogenous CO production.

eCO measurement was possible in all of the 21 EVLP procedures and, as expected, did not perturb their progress. The ProCeas allows monitoring in real-time eCO during the entire EVLP as plotted in Figure 2, where four recruitments periods, spaced by inter-recruitment periods, were performed. The thickness of the eCO trace in this figure corresponds to the concentration oscillations during inhalation and exhalation phases, as shown in the zoom-in Figure 3. Indeed, the short response time of the analyzer allows the respiratory cycles to be resolved.

Three grafts had, from the beginning of the EVLP procedure, extremely high eCO values compared to other lungs. The only difference we observed in their donors’ medical history is that blood had been found in the respiratory tract during bronchoscopy. No other difference was found during EVLP procedures. These three lungs have been transplanted in the same way by the surgical team with good clinical results in recipients. A subgroup analysis comparing clinical outcomes of patients transplanted with these hemorrhagic lungs versus other patients transplanted with non-hemorrhagic lungs did not show any statistical difference (Table 2).

**FIGURE 4:**
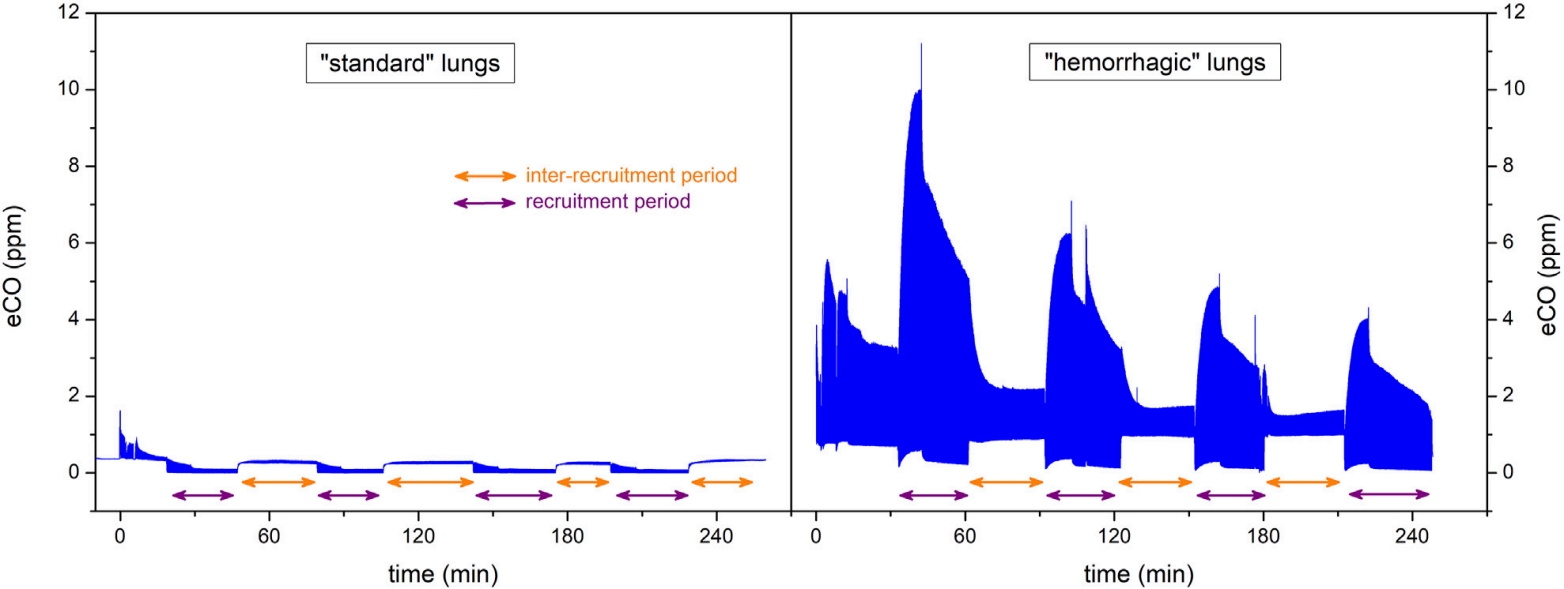
Data from two EVLP procedures displayed as examples of eCO monitoring of “standard” lungs (left) and eCO monitoring of “hemorrhagic” lungs (right). Among the total of 21 EVLP procedures that we monitored, only 3 pairs of lungs had an “hemorrhagic profile” like the one shown on the right part of this figure. Although these 3 pairs of lungs were transplanted by the surgeons without problems for the recipients, we decided to exclude a priori these 3 pairs of lungs from the eCO data analysis.

Because of the very high concentrations of CO, which we assume to occur due to the presence of blood in the airways, we decided to exclude these three “hemorrhagic” paired grafts from the eCO data analysis. They were excluded *a priori* (prior to commencement of the data analysis). An example of the eCO measurements of these hemorrhagic lungs is given in Figure 4.

There was no difference in eCO levels depending on the origin of the marginal grafts (Brain Dead Donors or DCD), for this reason we did not make a subgroup for the analyses.

**FIGURE 5:**
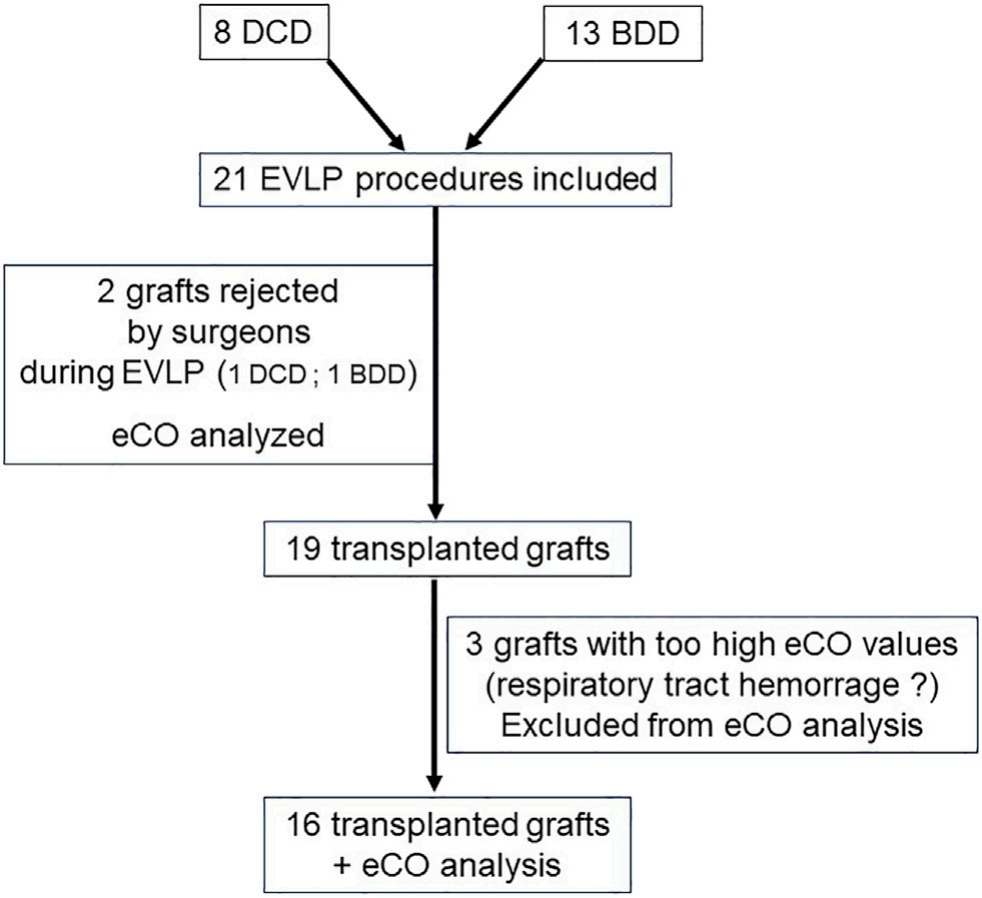
Flow chart. Abbreviations: DCD: Donor after Circulatory Death, BDD: Brain Dead Donors.

The analysis flow chart is presented in Figure 5.

### Acceptance of Grafts

Table 3 displays eCO levels in the 18 lungs where eCO could finally be analyzed. At the end of the 1st recruitment maneuver, eCO value in the two non-accepted lungs for transplant was 0.358 (±0.051) ppm. In the 16 other transplanted lungs, eCO value was 0.240 (±0.076) ppm. There was a tendency towards higher eCO levels in non-accepted lungs (*p* = 0.068).

**TABLE 3:** eCO at each recruitment phase in the 18 EVLP where eCO could finally be analyzed.

N°	eCO end 1st recruitement	eCO end 2nd recruitement	eCO end 3rd recruitement	eCO end 4th recruitement
1	0.139	0.115	0.101	
2	0.102	0.094	0.089	0.078
3	0.047	0.065	0.062	
4	0.114	0.104		
5	0.278	0.256		
6	0.306	0.411		
7	0.409	0.19		
8	0.218	0.229	0.165	
10	0.081	0.102	0.101	0.091
12	0.041	0.046	0.041	0.04
13	0.125	0.116	0.117	
15	0.235	0.2	0.167	
16	0.163	0.194	0.164	
17	0.159	0.143	0.105	0.091
18	1.338	1.084	0.94	
19	0.363	0.313	0.305	
20	0.27	0.294	0.105	0.242
21	0.163	0.13	0.112	0.096

In grey, the two non-accepted lungs for transplant after EVLP, eCO values of the 3 excluded procedures are not shown, eCO end 1st R = mean of the eCO during the 5 last minutes of the first recruitment phase etc. Values given in ppm.

The lungs 9, 11, and 14, excluded from the eCO analysis (macroscopical blood in their respiratory tract), are not presented in this table.

At the end of 1st recruitment: eCO value in the two non-accepted lungs for transplant was 0.358 (±0.051) ppm. In the 16 other transplanted lungs, eCO value was 0.240 (±0.076) ppm.

There was a tendency towards higher eCO levels in non-accepted lungs (*p* = 0.068).

### Parameters Tested During the *Ex-Vivo* Lung Perfusion

There was a tendency for higher vascular resistance for lungs with high eCO levels (*p* = 0.062). However, there was no association between eCO levels and gas exchange capacities, pulmonary compliances, nor a decrease in perfusion fluid in the EVLP reservoir (as a reflection of pulmonary edema formation).

**FIGURE 6:**
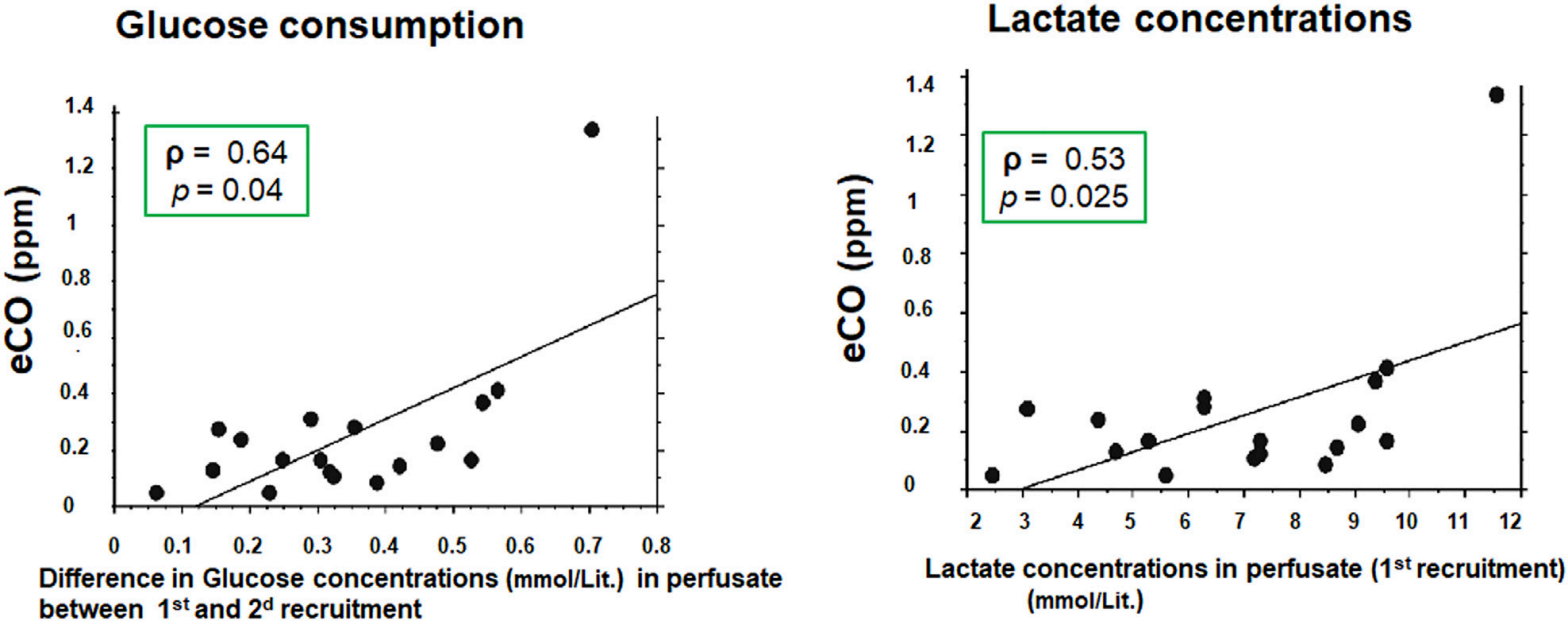
eCO and metabolism. Glucose consumption is estimated as the difference in glucose concentration in perfusate between the two first recruitment maneuvers. When removing the outlier point (lung #18), the correlation is still significant (*p* < 0.05; ρ = 0.49) between eCO and glucose consumption, but not anymore with lactate concentration.

There was a positive correlation between eCO and glucose consumption estimated by the difference in glucose levels in two perfusate samples taken 1 hour apart (*p =* 0.042). ECO was also correlated with lactate levels in perfusate (*p =* 0.035) (Figure 6).

### Short-Term Outcomes of the Recipients

There was a strong tendency (*p* = 0.051; *ρ* = 0.495) for a negative correlation between eCO after the first recruitment maneuver of the EVLP and PaO_2_/FiO_2_ ratio in recipients 24 h after transplantation.

A Receiver Operating Characteristic (ROC) curve has been drawn to assess the value of eCO and to predict an alteration in post-operative gas exchange (PaO_2_/FiO_2_ < 300 mmHg, 24 h after transplantation). The Area Under the Curve (AUC) was 0.668 and the most relevant threshold of eCO was 0.235 ppm (Youden index [Se + Sp-1] = 0.444). Using this threshold of 0.235 ppm, lung grafts were separated into two groups: lungs exhaling high eCO levels (>0.235 ppm) and those exhaling low eCO levels (≤0.235 ppm) after the first recruitment maneuver of the EVLP. Every patient who received grafts exhaling high eCO levels during the EVLP had a PGD score of 3 within 72 h. Conversely, only one patient with a low eCO level graft had a PGD score at 3 in 72 h.

**FIGURE 7:**
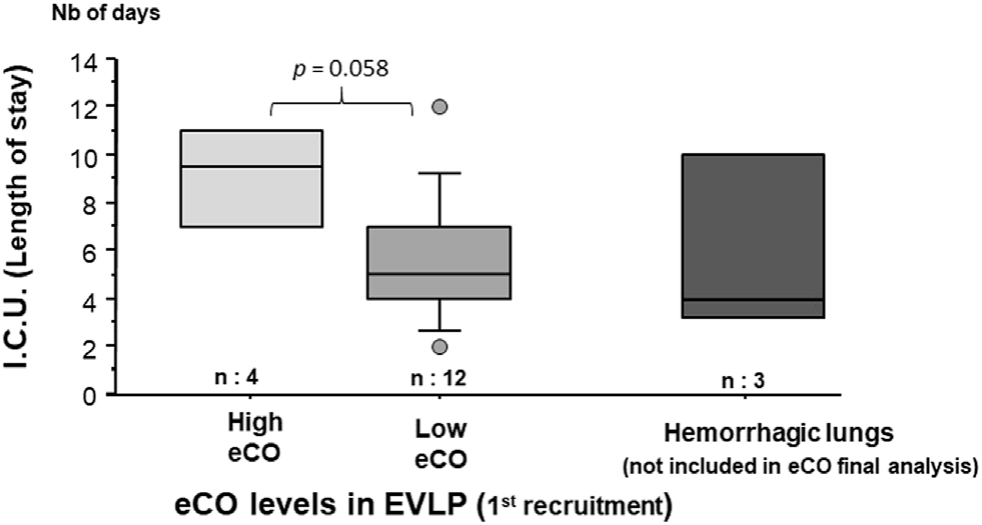
eCO and length of stay in ICU. Patients receiving lungs exhaling “high” eCO levels (>0.235 ppm) at 1st EVLP evaluation, have a trend to stay longer in ICU.

There was a trend towards a longer stay in intensive care unit for patients who received grafts with high eCO levels during EVLP (*p* = 0.058) (Figure 7). All patients transplanted with an EVLP lung were alive at 30 days.

### Inflammatory Proteins

There was no significant correlation between cytokines concentrations in perfusion fluid and eCO levels during the EVLP. Nevertheless, there was a positive correlation between TNF-*α* glucose consumption (*p* = 0.033), and lactate production (*p* = 0.049) as well as between IL-1β and PaO_2_/FiO_2_ ratio in recipients 24 h after transplantation (*p* = 0.039), duration of the mechanical ventilation (*p* = 0.002) and length of time in ICU (*p* = 0.004).

## Discussion

Our study confirms the feasibility and the accuracy of OF-CEAS non-invasive continuous monitoring of exhaled CO in EVLP lung grafts. In our series, despite the very low number (2) of lungs rejected for transplantation after the EVLP, eCO shows interesting trends for a future marker of lung graft status. A significant amount of blood into airways may, however, interfere with the eCO measurement.

### Limitations of the Study

This was a single-center study, the first objective of which was to assess the feasibility of eCO measurement by ProCEAS® in EVLP applied to human lung. The number of EVLP procedures performed over a seven- month period of observation is the highest published in Europe at that time. However, statistically speaking, this is a relatively small number, and only two grafts were recused for transplantation after the EVLP. Therefore, our observations about the eCO as an early marker of acceptance for the graft or as a prognostic marker for the receivers need to be interpreted with caution. The next step, to increase the statistical power of the evaluation of eCO for EVLP, will be to set up a multicenter study which will probably have to be international, given the few centers mastering the EVLP technique with a sufficient rate of procedures. Another limitation of the technique is in case of presence of blood in the airways. The degradation of the heme contained in large quantities in the blood is responsible for the production of CO in large quantities and has been widely described in the literature (22, 23). This probably explains the extremely high CO levels observed in grafts for which blood was visualized by bronchoscopy.

### Influence of Ventilatory Parameters on the eCO Measurements

The ventilator of the XPS™ device uses mixed gases (medical O_2_ from operating room gas supply and ambient air). Except during recruitment maneuvers where lungs were ventilated with pure oxygen, the EVLP procedure was performed all the time at 21% FiO_2_ using ambient air. Ambient air contains CO from air pollution and the breathing of nursing staff. Therefore, at 21% FiO_2_, the minimum of eCO, measured during the inspiratory phase reflected the CO contained in ambient air (Figure 2). This intermittent intake of external CO explains the staircase aspect of the eCO curves. However, the cycle-to-cycle measurement of eCO made it possible to overcome these variations in the baseline (Figure 3). The increase to 100% FiO_2_ (with pure oxygen without any CO) at the beginning of each recruitment phase showed a drop near zero of the eCO bottom line (inspiratory phase), while displaying a huge peak of eCO at the expiratory phase. As an increased PEEP to 10 cmH2O was applied at the same moment for recruitment, it is difficult to figure out the respective influence of each setting on eCO variation. Probably the highest PEEP led to a transient increase of eCO due to a better alveolar recruitment, allowing the elimination of CO, having no influence, however, on its production. On the other hand, a high concentration of oxygen may have been responsible for a competitive effect on CO adsorption on heme or other metalloproteins, as previously described (17, 24, 25) and may explain a part of the increase in eCO.

While lowering PEEP back to 5 cmH2O at the end of the recruitment phase, eCO dropped to lower values but reached a very stable phase (Figure 3) which probably represents the balance between newly recruited alveoli and previously aerated areas. The evaluation phase (blood gases, hemodynamics, eCO measurement averaged over 1 minute) took place at this steady state (left graph in Figure 3). The return in ambient air containing CO (end of the evaluation phase) led to an increase in eCO bottom line (inspiratory phase) and an irregular eCO exhalation in the expiratory phase. This probably reflects the end of hyperoxia effect on CO adsorption on metalloproteins.

### Comparison With Other Markers During the *Ex-Vivo* Lung Perfusion

Our data showed a correlation between eCO and both glucose consumption as well as lactate levels in the perfusate. Valenza et al. have already shown that the consumption of glucose during an EVLP in pig lungs was correlated with pulmonary edema (26). Effros et al. demonstrated that fluid reabsorption in edematous rat lungs increased glucose consumption (27). The stimulation of alveolar fluid clearance, by β-Adrenergic agonist infusion, increased glucose consumption in an EVLP pig lung model (28). In our series, it is conceivable that injured lungs struggled against the development of alveolar edema by activating the Na/K/ATPase pump system thus increasing alveolar fluid clearance. The energy cost of Na-K pump activity in an anaerobic environment could explain the increased glucose consumption and lactates levels in injured lungs exhaling high concentrations of eCO and expressing high levels of pro-inflammatory cytokine TNF-α.

The lack of correlation between eCO levels and PO_2_/FiO_2_ during the EVLP may be due to a delay between gas exchange alterations compared to the onset of inflammatory ischemia-reperfusion lesions. Indeed, Yeun et al. have shown, in a porcine EVLP model, that *ex vivo* PO_2_ may not be the first indication of lung injury in cell-free EVLP models and, taken alone, may be misleading in assessing the *ex vivo* lung (29). This argues in favor of a more reliable marker of pulmonary lesion in EVLP.

### eCO and Recipients’ Outcomes

CO is a gasotransmitter known to have anti-inflammatory, anti-apoptotic, anti-proliferative, and anti-thrombotic properties by the activation of soluble guanylate cyclase (sGC) and cyclic guanosine monophosphate (cGMP) pathways (9). In pig and rodent ischemia-reperfusion models, lungs administered with exogenous CO showed less histologic damages, lower levels of inflammatory cytokines (IL6 and TNFalpha), and had much better gas exchange capacities (30–32). Therefore, grafts exhaling high eCO levels might be those with a high potentiality to react and adapt to ischemia-reperfusion injury. However, our results do not support this hypothesis and a high eCO level seems to be associated with a poorer outcome of the grafts. In our series, elevated eCO (>0.235 ppm) at the first EVLP recruitment procedure predicted a PGD of 3 within the first 72 h post-transplantation. Therefore, high eCO levels could warn about difficult short-term outcomes of the recipients and the need for sustained supportive care. Conversely, in low eCO grafts, better outcomes might be expected and, for example, earlier extubation could be considered.

## Conclusion

During EVLP, eCO can be measured continuously and non-invasively thanks to the ProCEAS® analyzer. It appears to be associated with the severity of the ischemia-reperfusion injury and could provide new information for early acceptance of transplants. Future multicenter studies about eCO and EVLP are necessary to provide stronger evidence.

## Data Availability

The datasets presented in this study can be found in online repositories. The names of the repository/repositories and accession number(s) can be found in the article/Supplementary Material.

## Abbreviations

BDD, brain dead donors; CO, carbon monoxide; eCO, endogenous exhaled carbon monoxide; DCD, donor after circulatory death; ECMO, extra corporeal membrane oxygenation; EVLP, *ex-vivo* lung perfusion; HO, heme oxygenase; IL, interleukine; ISHLT, International Society for Heart and Lung Transplantation; OF-CEAS, optical-Feedback cavity-enhanced absorption spectroscopy; PGD, primary graft dysfunction; ppm, parts per million; TNF, tumoral necrosis factor.
